# Spatial Association of Canine Rabies Outbreak and Ecological Urban Corridors, Arequipa, Peru

**DOI:** 10.3390/tropicalmed2030038

**Published:** 2017-08-13

**Authors:** Ricardo Castillo-Neyra, Edith Zegarra, Ynes Monroy, Reyno F. Bernedo, Ismael Cornejo-Rosello, Valerie A. Paz-Soldan, Michael Z. Levy

**Affiliations:** 1 Department of Biostatistics and Epidemiology, Perelman School of Medicine, University of Pennsylvania, Philadelphia, PA 19104, USA; mzlevy@mail.med.upenn.edu; 2 Zoonotic Disease Research Lab, One Health Unit, School of Public Health, Universidad Peruana Cayetano Heredia, Lima 15102, Peru; 3 Gerencia Regional de Salud de Arequipa, Ministerio de Salud, Arequipa 04002, Peru; zegarraedith@gmail.com (E.Z.); yni1923@hotmail.com (Y.M.); florentino39@hotmail.com (R.F.B.); quilco2005@yahoo.es (I.C.-R.); 4 Department of Global Community Health and Behavioral Sciences, Tulane University School of Public Health and Tropical Medicine, New Orleans, LA 70112, USA; vpazsold@tulane.edu

**Keywords:** dogs, geographical information system, GIS, Monte Carlo method, rabies, spatial analysis, zoonosis

## Abstract

In the city of Arequipa, Peru, a rabid dog was detected in March 2015, marking the reintroduction of the rabies virus in the area; more rabid dogs have been detected since then. The presence of free-roaming dogs in Arequipa seems to be higher in dry water channels, which are widespread in the city. We created a geographic information system (GIS) with surveillance data on the location of rabid dogs detected during the first year of the outbreak, as well as the water channels. We conducted a spatial analysis using Monte Carlo simulations to determine if detected rabid dogs were closer to the water channels than expected. Thirty rabid dogs were detected during the first year of the outbreak, and they were statistically associated with the water channels (average distance to closest water channel = 334 m; *p*-value = 0.027). Water channels might play a role in the ecology of free-roaming dog populations, functioning as ecological corridors. Landscape ecology could assist in understanding the impact of these urban structures on control activities and the persistence of transmission.

## 1. Introduction

The reintroduction of canine rabies to areas declared free of the virus is a rare event globally [1,2,3,4]. The outbreak of canine rabies in Arequipa, Peru in March 2015 is the first instance of canine rabies reintroduction in Latin America, where enormous advances have been achieved through dog vaccination [5]. Fortunately, no human cases have been detected in Arequipa to date. Genetic sequencing suggests that the rabies virus was introduced to Arequipa by dogs imported from Puno, a neighboring region in southern Peru that has reported sustained transmission of canine rabies for the last 15 years [6]. Interestingly, the urban landscape in Arequipa is characterized by the presence of large open water channels that are dry most of the time, where free-roaming dogs can be found.

**Figure 1:**
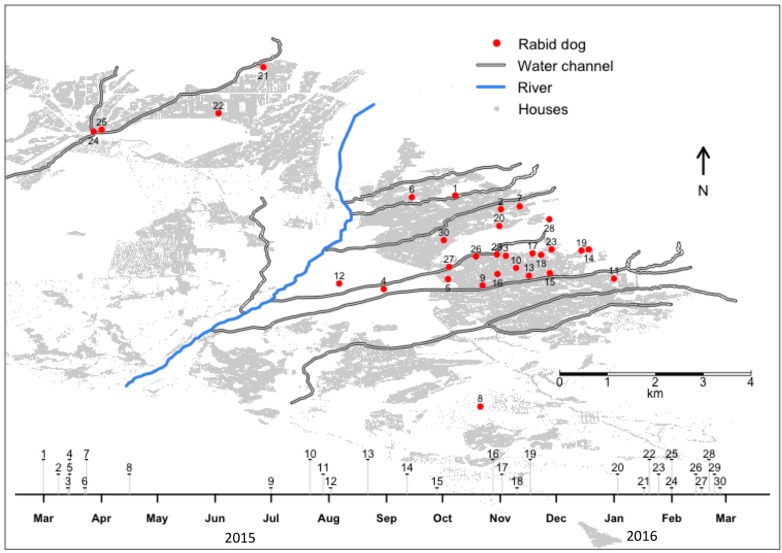
Spatio-temporal distribution of laboratory-confirmed rabid dogs and water channels system in the city of Arequipa, March 2015–March 2016 (different heights in timeline are used only to avoid overlap).

In Arequipa, where 22% of owned dogs have free access to the street all the time, and 48% of owned dogs are unrestricted at some point during the day [7], it is unknown which proportion of dogs seen on the streets are actually stray dogs and which are owned. Upon detection of the first rabid dog, Ministry of Health (MOH) authorities attempted containment by ring vaccination of an approximately five-block radius, in accordance with the Peruvian technical guidelines to control and prevent human rabies [8]. In addition, they conducted contact tracing: dogs that lived within that approximate five-block radius and that could be considered potentially exposed to the virus (unvaccinated and with relatively free access to the street or known history of contact with free-roaming dogs) were euthanized [8]. Humans potentially exposed to the virus were offered post-exposure prophylaxis [8]. Days later, a second case was detected in another district, over 1.25 kilometers from the original case (Figure 1); the same containment strategy was implemented. The next case appeared in still another district, and each subsequent case continued to be detected far beyond the containment area of the last (Figure 1). Four months after the first case was detected, mass dog vaccination campaigns were implemented by the MOH. Despite the initiation of this city-level approach in 2015, ring containment immediately following the detection of a case has also been practiced until 2017. The median vaccination coverage reported among the 29 districts of the province of Arequipa was 101% in the first year of the outbreak, with three districts reporting coverage above 200%. Similarly, the reported coverage in the ring vaccination activities was higher than 100% in all cases, suggesting a substantial underestimation of the dog population at that time. Additionally, culling of free-roaming dogs (without evidence in favor of this strategy [9,10,11]) has been conducted by some groups.

The reemergence of the virus transmission in Arequipa has been associated with the high density of free-roaming dogs in the city (i.e., stray and owned dogs that spend unsupervised time in the streets and water channels), and with low dog vaccination coverage [12]. The authorities report that water channels are central to the problem, because the density of dogs is higher in them, dogs feed on trash disposed in the channels, and large packs of dogs have been seen moving along these urban structures. In other parts of the world, urban structures similar to the dry water channels in Arequipa are used by wild animals to move within city matrices. Foxes use ravines to move across urban areas of Toronto [13], and bobcats and coyotes use culverts and linear fragments of vegetation to circulate in southern California [14]. Here we considered the dry water channels of Arequipa as ecological corridors, following the definition given by Freemark for corridors [15]: “a physical linkage between habitat patches within a landscape that may serve as a pathway by which organisms move or interchange, or as a habitat in which organisms can feed or breed en route from one patch to another.” These urban ecological corridors and other physical aspects of urbanization in fast-growing cities such as Arequipa facilitate the emergence and persistence of zoonotic and emergent disease [16,17,18] and may complicate canine rabies control.

Despite the efforts to control the outbreak, transmission of the rabies virus continues in the city of Arequipa, placing the approximately 1 million inhabitants of the city at risk of infection. The main objective of our study was to assess the spatial association between detected rabid dogs and the most salient urban structures in the city of Arequipa—the dry water channels.

## 2. Materials and Methods

### 2.1. Study Setting

**Figure 2:**
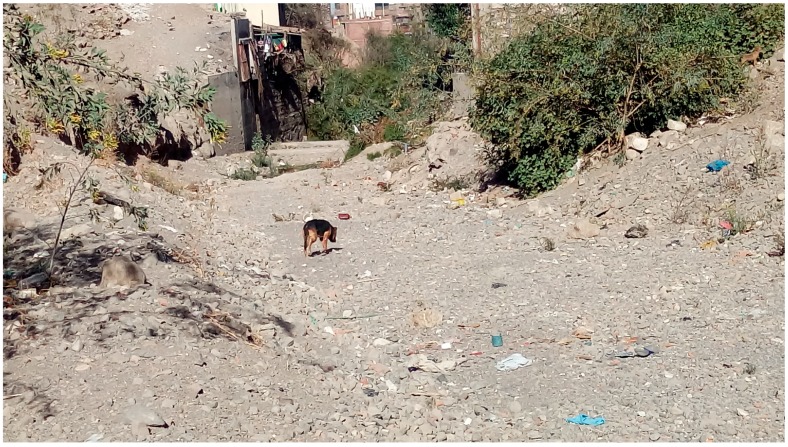
Dog in a dry water channel in the city of Arequipa.

The city of Arequipa is located in the Andes at 2300 m above sea level; its temperature ranges from 10 °C to 29 °C. The Chili River runs through the city, and rainwater drains to it via uncovered semi-natural water channels. The rainy season lasts 8 weeks; the channels are dry the remainder of the year (Figure 2). The city of Arequipa has grown significantly during recent decades, reaching a population of approximately 998,000 people in 2015 and covering an urban area of over 101 square kilometers. On the outskirts of the city, urbanization creates peri-urban areas that, after decades, are absorbed by the continuous expansion of the city [18]. Arequipa is surrounded by desert, which has little wildlife that could sustain the rabies virus, nor resources (shelter, food) to support feral dog populations.

### 2.2. Data

As part of routine surveillance activities, the MOH Reference Laboratory in Arequipa tested brain samples of suspected rabies cases using direct fluorescent antibody [19]. Additionally, mouse inoculation testing [19] was conducted at the National Institute of Health Laboratory in Lima. Samples positive to either test were considered rabies positive. We created a geographic information system (GIS) with the coordinates of all the houses in the city of Arequipa, as well as those of the system of water channels. Over these geographic layers, we added the location of the canine rabies cases detected during the first year of the outbreak (March 2015 to March 2016). The rabies surveillance system in Peru only acquires location data of the health facility catchment where the sample was collected (areal data). Our team investigated the exact point location of positive samples. Some of these point locations were the house of the reporting owner, or the spot where a dead dog was found or an aggressive or disoriented dog was captured. We report the number of samples analyzed and why they were submitted for diagnosis.

### 2.3. Statistical Analysis

We conducted a spatial analysis to test the hypothesis that rabid dogs were detected closer to a water channel than would be expected by chance. We calculated the average shortest Euclidian distance between the location of rabid dogs and the closest water channel. The exact coordinates of negative dogs were not available. We used a Monte Carlo random-labeling simulation with 1000 trials to compare the average distance between observed rabid dogs and water channels to the distance from water channels of a randomly-generated spatial sample of households [20], matched on locality. We have found that the number of owned dogs per house can vary by locality [7], and we also wanted to take into account any unobserved difference in surveillance intensity at the locality level. Localities are informal district subdivisions which are widely used by the health inspectors to differentiate their catchment areas and organize their work. Under the null hypothesis, we assumed that all households are equally likely to have a rabid dog regardless of their distance to the water channels—on a door-to-door survey conducted in more than 4000 houses by our team, we did not find an association between the number of owned dogs per house and the water channels [7]. To determine whether the observed distance of rabid dogs to water channels was statistically different from the distance of the water channel to the random spatial sample, we compared the average observed distance to the distance distribution derived from the Monte Carlo trials. The proportion of distances in the Monte Carlo-generated random sample which were lower than the average observed distance was the estimate Monte Carlo *p*-value.

Separately, we assessed the spatial clustering of rabid dogs using the L statistic [20]. Briefly, spatial clustering is defined as a general tendency for point pattern events (here, detected rabid dogs) to occur more closely together than would be expected under complete spatial randomness (CSR) [21]. We also estimated the distance between each case and the subsequent case. Finally, we present if detected cases were owned dogs, and if it was reported that they bit any humans. The analyses were conducted in R [22].

## 3. Results

Out of 559 samples analyzed between 17 March 2015 and 16 March 2016, only 397 of the analyzed samples had some data about the case in the reporting form: 77 samples came from dogs hit by a car, 55 samples were collected during ring-containment activities, 43 forms reported that the dog was found dead or dying outdoors, 41 samples reportedly came from aggressive dogs, 37 samples came from dogs with neurological signs, 25 dogs were reported as “sick” or “sick and died”, 21 samples came from dogs that were killed or euthanized by their owners, 18 came from dogs found in the water channels, and 11 were samples or dogs sent by private veterinarians. Sixty-nine (69) forms state that the dog was found outside or was stray, but do not mention if the dog was dead when found. Thirty (30) dogs were diagnosed positive for rabies (5.37%) (Figure 1); 13 of the positive dogs were first suspected cases because they were aggressive, one was the contact of a positive dog, one was sent by a private veterinarian, one had clinical signs compatible with rabies, one was captured because it was a stray dog, and one was sent to the lab because it was “sick”.

**Figure 3:**
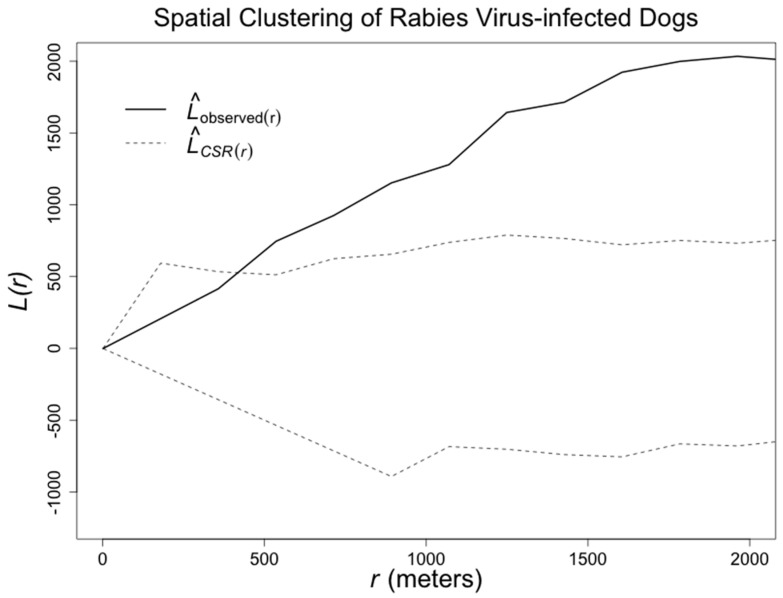
Strong clustering of rabid dogs evaluated with the L function. Envelopes (dashed lines) produced assuming complete spatial randomness (CSR).

The average distance between detected rabid dogs and water channels was 334 m. In only 27 of the 1000 Monte-Carlo simulated location sets was the average distance to the water channels 334 m or lower (Monte Carlo *p*-value = 0.027). When analyzing all the detected cases together, we found significant spatial clustering of confirmed rabid dogs: overall, cases were detected more closely together than we would expect at random (Figure 3). However, some of these cases that were close to each other were detected several months apart. The median distance between each detected case and the next case was 2.253 km, and 86.7% of the cases were detected one km or farther from the previous case. Importantly, at least 43% of the rabid dogs in this study had bitten at least one human and 76% of the rabid dogs were turned in to authorities by their owners. These percentages likely underestimate the actual percentages of dog bites by rabid dogs and dog ownership among detected rabid dogs.

## 4. Discussion

Rabies-virus positive dogs are spatially associated with water channels in the city of Arequipa. This association is unlikely to be related to sampling bias, as the rabies surveillance program is mostly passive: the majority of rabid dogs detected during this outbreak were reported by their owners. Free-roaming dog ecology and rabies virus transmission (e.g., habitat, movement, interactions) could be strongly influenced by water channels, as these urban structures—where trash is commonly dumped by dwellers—can produce an “ecological assembly” [23] within the city, increasing the density of dogs. Water channels may also form ecological corridors [24], increasing the connectivity of spatially-distant dog populations. It is possible that by increasing the connectivity of dog packs, the water channels facilitate the persistence of the virus by allowing it to reach new susceptible packs, similar to what has been observed at larger scales in raccoon rabies [25]. 

In the city of Arequipa, the high spatial heterogeneity created by the water channels poses challenges to modeling contact and networks that capture the spatial dog population structure, and analyzing models that include long-distance interactions between individuals or packs [26]. In addition, what facilitates dog movement could impede human movement: the water channels are barriers for pedestrians and could decrease access to vaccination points, thereby creating pockets of low canine vaccination coverage, which could enable the persistence of rabies virus [27].

Clearly, containment activities have not controlled the outbreak; cases have been reported regularly through June 2017 in spatially disparate areas. A first look at the spatial distribution of cases could lead to the determination that cases are generally close to each other, which is also captured by the clustering analysis. The clustering analysis simply suggests that cases are not dispersed randomly all over the city. However, the distance between each case and the next detected case suggests that subsequent cases are not occurring close—or at least not close enough—to support focalized containment activities; thus, a citywide response is needed. The initial focalized containment strategy may have diverted resources [27] and delayed population-level responses such as mass dog vaccination [27] that have proven to be successful in other settings, but are not reaching the appropriate coverage levels [5,28,29,30] in Arequipa [31]. It is worth highlighting that Davlin and VonVille, in a systematic review on canine rabies vaccination, concluded that understanding of the local dog population ecology is important to achieve effective vaccination coverage [32]. The unrealistic vaccination coverage estimates reported during the first year of the outbreak reflect inaccurate estimates of the dog population in the city of Arequipa. The human-to-dog ratio used to estimate the dog population was 10:1 in 2015; that ratio has been reduced to 6:1 for 2016 and 2017, approaching the true ratio, but a better knowledge of the canine population is still necessary.

In Arequipa, there are a number of plans to control the canine population that is found in the water channels. To some extent, these plans recognize the spatial heterogeneity produced by urban structures. However, if plans include the culling of free-roaming dogs, there may be negative unintended consequences [10,11,33,34,35,36]. In addition, these measures may not take into account the potential functionality of these ecological urban corridors [24] on free-roaming dog ecology. Additionally, these water channels serve a political function: they delineate boundaries between districts, which determine jurisdictions for rabies health inspectors. These areas within the water channels become a no-man’s-land in the surveillance system; dead dogs are left uncollected and undiagnosed. A centralized surveillance system at the city level could better address these areas that seemingly do not belong to any district jurisdiction. The design of urban landscapes has received much attention around the world to improve health outcomes. However, most of this attention is focused on chronic disease, mental health, violence and injuries, and pollution [37]. Landscape ecology has the potential to be used in urban areas to understand zoonotic diseases and guide land management, as it has been applied for rabies in wild and rural areas [25,38,39,40,41,42].

One limitation of our study was the lack of locations for negative dogs—more detailed data accompanying the samples sent to the laboratory would allow for analyses with fewer assumptions. Related to the surveillance system, it is important to note that surveillance systems that are based on meeting a pre-defined quota of samples frequently receive and analyze samples of non-suspicious animals and miss the vast majority of cases [43]; therefore, the 30 rabies-positive dogs detected during the first year of this outbreak most likely represent only a fraction of the total number of cases. New surveillance systems based on active rabid-dog finding guided by triage data from dog-bite patients has the potential to increase the probability of case detection by several orders of magnitude [43]. Given that a great extent of cases were most likely missed, there is the possibility that our results of spatial clustering may be different if it were possible to account for those cases not detected by the surveillance system. It is also possible that other less ecological and more social phenomena are explaining the association between cases and water channels: dog vaccination status could be lower around the water channels due to spatial differences in educational attainment, economics, logistical constraints, among other factors. Dog ownership practices also affect dog ecology: households closest to the water channels may restrain their animals less, which would result in an increase of the free-roaming dog population in the water channel areas. Further investigations on vaccination patterns, the urban landscape, and the ecology of dogs in Arequipa could inform surveillance and control activities.

## Supplementary Materials

The following are available online at www.mdpi.com/2414-6366/2/3/38/s1, S1 Abstract. Resumen en español (alternative language abstract).
