# Supplementary material for: Spatial Association of Canine Rabies Outbreak and Ecological Urban Corridors, Arequipa, Peru
Source: Trop Med Infect Dis. 2017 Aug 13;2(3):38. doi: 10.3390/tropicalmed2030038 (PMC6082090; doi:10.3390/tropicalmed2030038)
Supplement: Supplementary file 1 [file tropicalmed-02-00038-s001.pdf]

Supplementary Materials: Spatial Association of Canine Rabies Outbreak and Ecological Urban Corridors, Arequipa, Peru

Ricardo Castillo-Neyra, Edith Zegarra, Ynes Monroy, Reyno F. Bernedo, Ismael Cornejo-Rosello, Valerie A. Paz-Soldan and Michael Z. Levy

S1 Abstract. Resumen en español (Alternative language abstract).

En la ciudad de Arequipa, un perro con rabia fue detectado en marzo del 2015, marcando la reintroducción del virus de la rabia en el área; más perros con rabia se han detectado desde entonces. La presencia de perros vagabundos, con y sin dueño, en Arequipa parece ser mayor en los canales de agua secos, los cuales están muy extendidos por la ciudad. Creamos un sistema de información geográfica (SIG) con datos de vigilancia sobre la ubicación de los perros con rabia detectados durante el primer año del brote y los canales de agua. Realizamos un análisis espacial utilizando simulaciones Monte Carlo para determinar si los perros rabiosos detectados están más cerca de los canales de agua de lo esperado. Se detectaron treinta perros con rabia durante el primer año del brote y estos estaban estadísticamente asociados a los canales de agua (distancia promedio al canal de agua más cercano = 334 m, valor de p = 0,027). Los canales de agua podrían desempeñar un papel en la ecología de las poblaciones de perros vagabundos y funcionar como corredores ecológicos. La ecología del paisaje podría ayudar a comprender el impacto de estas estructuras urbanas en las actividades de control y la persistencia de la transmisión.
